# The clinical impact of serum soluble CD25 levels in children with Langerhans cell histiocytosis

**DOI:** 10.1016/j.jped.2024.08.005

**Published:** 2024-09-10

**Authors:** Zi-Jing Zhao, Hong-Yun Lian, Wei-Jing Li, Qing Zhang, Hong-Hao Ma, Dong Wang, Yun-Ze Zhao, Ting Zhu, Hua-Lin Li, Xiao-Tong Huang, Tian-You Wang, Rui Zhang, Lei Cui, Zhi-Gang Li

**Affiliations:** a Hematologic Diseases Laboratory, Beijing Pediatric Research Institute, Beijing Children's Hospital, Capital Medical University, National Center for Children’s Health, Beijing, China; b National Key Discipline of Pediatrics, Capital Medical University, Beijing, China; c Key Laboratory of Major Diseases in Children, Ministry of Education, Beijing, China; d Department of Hematology, Hematology Center, Beijing Children's Hospital, Capital Medical University, National Center for Children’s Health, Beijing, China

**Keywords:** Langerhans cell histiocytosis, sCD25, Prognosis, Relapse

## Abstract

**Objective:**

Langerhans cell histiocytosis (LCH) is a rare myeloid neoplasm with inflammatory characteristics. This study aims to investigate the correlation between sCD25 levels and clinical characteristics, as well as prognosis, in pediatric LCH.

**Methods:**

Serum sCD25 levels were measured in 370 LCH patients under 18 years old using ELISA assays. The patients were divided into two cohorts based on different treatment regimens. We further assessed the predictive value for the prognosis impact of sCD25 in a test cohort, which was validated in the independent validation cohort.

**Results:**

The median serum sCD25 level at diagnosis was 3908 pg/ml (range: 231–44 000pg/ml). sCD25 level was significantly higher in multi-system and risk organ positive (MS RO^+^) LCH patients compared to single-system(SS) LCH patients (*p* < 0.001). Patients with elevated sCD25 were more likely to have involvement of risk organs, skin, lung, lymph nodes, or pituitary (all *p* < 0.05). sCD25 level could predict LCH progression and relapse, with an area under the ROC curve of 60.6 %. The optimal cutoff value was determined at 2921 pg/ml. Patients in the high-sCD25 group had significantly worse progression-free survival compared to those in the low-sCD25 group (*p* < 0.05).

**Conclusion:**

Elevated serum sCD25 level at initial diagnosis was associated with high-risk clinical features and worse prognosis. sCD25 level can predict the progression/recurrence of LCH following first-line chemotherapy.

## Introduction

Langerhans cell histiocytosis (LCH) is the most common histiocytic disorder, encompassing a broad range of clinical manifestations and outcomes, from self-limited lesions to life-threatening disseminated disease.^1^^,^^2^ Over the past decade, recurrent somatic activating gene mutations in the mitogen-activated protein kinase (MAPK) pathway have been identified in approximately 85 % of LCH lesions.^3^^,^^4^ Further research has defined LCH as an inflammatory myeloid neoplasia, with the extent of disease corresponding to the cell of origin in which activating mutations arise.^5^ LCH lesions contain Langerhans cells (CD1a^+^/CD207^+^ dendritic cells) alongside a prominent inflammatory infiltrate of various immune cells (T cells, macrophages, eosinophils, neutrophils, and natural killer cells, etc.), which also contribute to aspects of pathogenesis.^6^^,^^7^ These infiltrating cells produce large amounts of pro-inflammatory cytokines and chemokines, creating a lesional cytokine storm.^8^ Several studies have shown that increased serum levels of cytokines and chemokines, such as IL-6, IL-10, IL-18 and TNF-α, were associated with the disease extent or the mutation status in LCH.^9^, ^10^, ^11^

Soluble CD25 (sCD25), a soluble form of the α-subunit of the interleukin-2 receptor (IL-2Rα), is generated exclusively by the proteolytic cleavage of the membrane-bound IL-2Rα, and its concentration is thought to reflect the immune activation during infection or inflammation.^12^ Elevated serum sCD25 has been detected in autoimmune inflammatory diseases, cancers, and infectious disorders, and it has been used as a biomarker of disease progression and prognosis.^13^, ^14^, ^15^, ^16^ Several studies have observed that pre-treatment sCD25 level was higher in LCH patients compared to healthy controls, and it significantly correlated with disease extent and survival of LCH.^17^ However, the clinical relevance and prognostic impact of sCD25 have not been fully clarified in pediatric LCH. In the present study, we retrospectively evaluated the correlation between sCD25 level and clinical-biological characteristics in pediatric LCH patients. We further assessed the predictive value for progression/relapse and prognosis impact of sCD25 in a test cohort, which was validated in an independent validation cohort.

## Material and methods

### Patients and cohorts

A total of 469 consecutive patients with newly diagnosed LCH (age < 18 years) were admitted to the center from January 1, 2017, to December 31, 2021. Of these, 370 patients with successfully determined sCD25 at the time of diagnosis were eligible for analysis in the study. Ninety-nine patients without sCD25 assessments were excluded. Subsequently, 359 patients who received first-line therapy were further analyzed with prognostic significance, leaving out 11 patients who directly received BRAF inhibitor dabrafenib treatment. We divided the 359 patients into two groups based on the year of enrollment: the test cohort comprised 146 patients enrolled in BCH-LCH 2014 clinical trial (www.chictr.org.cn, identifier: ChiCTR2000030457) between 2017 and 2018, while the validation cohort included 213 patients entered CCHG-LCH-2019 (www.chictr.org.cn, ChiCTR1900025783) between 2019 and 2021. A flow diagram for patient inclusion and the study cohorts is displayed in Supplementary Figure S1.

The diagnosis of LCH was confirmed through histological examination and CD1a and CD207 immunostaining. The following characteristics were collected from the electronic medical record system and LCH disease database for each patient: demographic information, clinical and biological characteristics, examinations, treatment details, and follow-up data.

This study was approved by the Beijing Childrenʼs Hospital Ethics Committee and was conducted in accordance with the Declaration of Helsinki. Informed consents were obtained from the guardians of the patients.

### Therapeutic regimen

Based on the number of involved organs/systems, LCH was categorized into two types: single-system (SS-LCH), which includes unifocal or multifocal involvement and multi-system (MS-LCH), which involves two or more organs/systems involvements. MS-LCH was further classified as risk organ (RO) positive (liver, spleen, and/or hematopoietic system) or negative based on the extent of LCH.^18^

Most of the enrolled patients received the standard first-line chemotherapy based on the LCH-III protocols.^19^ Briefly, the first-line therapy consisted of one or two six-week courses of initial induction therapy (vindesine-steroid combination) followed by maintenance therapy (vindesine, prednisone, with or without 6-mercaptopurine). Patients with involvement of non-central nervous system risk bones who responded well to the initial treatment were given six months of chemotherapy, while others received 12 months of overall treatment. The BRAF inhibitor dabrafenib was administered directly to *BRAF*-V600E-mutated MS-LCH patients who were under two years old or unable to endure the standard chemotherapy.^20^ Treatment responses were assessed according to the International LCH Study Group criteria.^7^^,^^18^

### Measurement of serum sCD25 and cytokine levels

Peripheral blood samples were collected from LCH patients at the time of initial diagnosis and one week after treatment in available patients. Serum samples were separated by centrifugation at 1000 g for 20 minutes within four hours of blood collection. Processed samples were either immediately analyzed or stored at −80 °C until use. Serum levels of sCD25 were measured in duplicate using a specific Enzyme-linked immunosorbent assay (ELISA; Kindstar Globalgene Technology Inc.). Serum cytokine levels of interferon-γ (IFN-γ), tumor necrosis factor α (TNF-α), IL-10, IL-6, IL-4 and IL-2 were determined by Cytometric Bead Array (Human Th1/Th2 Cytokine Kit II, BD Biosciences), as described previously.^21^

### *Detection of* BRAF-V600E *mutation in lesions and cfDNA*

We performed targeted DNA sequencing to detect mutations in LCH lesions using a customized genes panel (Supplementary Table S1) that covers 229 genes involved in MAPK, PI3K, or Jak/STAT pathways, Epithelial-mesenchymal transition, cell cycle, cell proliferation, apoptosis, cell adhesion/migration, and transcriptional regulation (MyGenostics Inc.).^22^ We determined *BRAF*-V600E levels in plasma cell-free (cf) DNA using the QX200TM System Droplet Digital PCR (ddPCR) System (Bio-Rad, Hercules, CA), as described previously.^23^

### Statistical analysis

Differences between groups were tested using the Kruskal–Wallis or Mann–Whitney *U* test for quantitative variables and Fisher's exact test for qualitative variables. Spearman's correlation test was used to determine the relationships. Receiver operating characteristic (ROC) curves were plotted to assess the efficacy of significant parameters and the optimal cutoff value was determined using the maximum Youden's index. A Bayesian formula was applied to analyze the sensitivity, specificity, positive predictive value (PPV), and negative predictive value (NPV) of the cutoff value. Progression-free survival (PFS) was estimated from the date of initial treatment until the date of one of the following events: progression, relapse, or death, whichever occurred first. The patients without events were censored at the date of the last contact. Univariate analysis for survival was performed using Cox regression to estimate the hazard ratio (HR). All risk factors with *p* < 0.05 in the univariate analysis were included as covariates in the Cox regression for multivariate analysis. A significant difference was defined as *p* < 0.05. Data were analyzed using SPSS 26.0 (IBM Corp, NY, USA) and R software (Version 4.2.1; R Foundation for Statistical Computing, Vienna, Austria). The cut-off date for follow-up was December 31, 2023.

## Results

### Clinical characteristics of LCH patients

The clinical characteristics of enrolled patients are shown in Table 1. The median age at diagnosis of LCH was 3.1 years. The ratio of boys to girls was about 1.6. Of the patients, 60.5 % of patients were SS LCH, 25.7 % had MS RO^−^ LCH, and 13.8 % had MS RO^+^ LCH. *BRAF*-V600E was the most common mutation, followed by MAP2K1 and *BRAF* other mutations.

**Table 1:** Clinical characteristics of enrolled patients with Langerhans cell histiocytosis.

Variables	n (%)	Patients with dabrafenib	Test cohort	Validation cohort	*P*-values^a^
Total patients	370	11	146	213	
Gender					
Male	230 (62.2)	5 (45.5)	88 (60.3)	137 (64.3)	0.439
Female	140 (37.8)	6 (54.5)	58 (39.7)	76 (35.7)	
Age (years)					
Median (age)	3.1 (0.1–15.1)	0.7 (0.1–2.1)	2.6 (0.1–11.6)	4.4 (0.1–15.1)	< 0.001
≥ 2	245 (66.2)	1 (9.1)	86 (58.9)	158 (74.2)	0.003
< 2	125 (33.8)	10 (90.9)	60 (41.1)	55 (25.8)	
Disease extents					
SS	224 (60.5)	0 (0)	84 (57.5)	140 (65.7)	0.106
MS RO^−^	95 (25.7)	4 (36.4)	38 (26.0)	53 (24.9)	
MS RO^+^	51 (13.8)	7 (63.6)	24 (16.4)	29 (9.4)	
Involvement					
Bone					
No	32 (8.6)	6 (54.5)	18 (12.3)	8 (3.8)	0.003
Yes	338 (91.4)	5 (45.5)	128 (87.7)	205 (96.2)	
Skin					
No	296 (80)	0 (0)	110 (75.3)	186 (87.3)	0.005
Yes	74 (20)	11 (100)	36 (24.7)	27 (12.7)	
Liver					
No	327 (88.4)	5 (45.5)	125 (85.6)	197 (92.5)	0.051
Yes	43 (11.6)	6 (54.5)	21 (14.4)	16 (7.5)	
Spleen					
No	343 (92.7)	6 (54.5)	133 (91.1)	204 (95.8)	0.077
Yes	27 (7.3)	5 (45.5)	13 (8.9)	9 (4.2)	
Hematologic					
No	344 (93.0)	6 (54.5)	135 (92.5)	203 (95.3)	0.264
Yes	26 (7.0)	5 (45.5)	11 (7.5)	10 (4.7)	
Lung					
No	311 (84.1)	6 (54.5)	120 (82.2)	185 (86.9)	0.233
Yes	59 (15.9)	5 (45.5)	26 (17.8)	28 (13.1)	
Lymph node					
No	329 (88.9)	9 (81.8)	131 (89.7)	189 (88.7)	0.864
Yes	41 (11.1)	2 (18.2)	15 (10.3)	24 (11.3)	
Pituitary					
No	350 (98.6)	7 (63.6)	138 (94.5)	205 (96.2)	0.447
Yes	20 (5.4)	4 (36.4)	8 (5.5)	8 (3.8)	
Ear					
No	347 (93.8)	11 (100)	134 (91.8)	202 (94.8)	0.276
Yes	23 (6.2)	0 (0)	12 (8.2)	11 (5.2)	
Eye					
No	359 (97.0)	11 (100)	142 (97.3)	206 (96.7)	1.000
Yes	11 (3.0)	0 (0)	4 (2.7)	7 (3.3)	
MAPK pathway gene mutations in tissue lesions					
Evaluable patients	311	11	105	195	
*BRAF*-V600E	172 (55.3)	11 (100)	62 (59.0)	99 (50.8)	< 0.001
*MAP2K1*	56 (18.0)	0 (0)	6 (5.7)	50 (25.6)	
*BRAF*-others	29 (9.3)	0 (0)	9 (8.6)	20 (10.3)	
Others	54 (17.4)	0 (0)	28 (26.7)	26 (13.3)	
*BRAF*-V600E in plasma cell-free DNA					
Evaluable patients	313	10	119	184	
Negative	209 (66.8)	0 (0)	72 (60.5)	137 (74.5)	0.011
Positive	104 (33.2)	10 (100)	47 (39.5)	47 (25.5)	

a *P* values for comparisons between the test cohort and the validation cohort.

A comparison of the test and validation cohort revealed no significant differences in most baseline characteristics, except for age, bone or skin involvements, and frequency of mutations. Eleven patients with *BRAF*-V600E mutations received dabrafenib treatment directly; seven were MS RO^+^ and four were MS RO^−^.

The 3-year PFS was 61.7 % ± 2.6 % for the entire cohort, with a median follow-up time of 33.4 months. Only one patient died of cirrhosis after treatment. The estimated median PFS time for the test and validation cohorts was 50.0 months and 42.7 months, respectively. Among the eleven patients treated with dabrafenib, six discontinued dabrafenib and four of them relapsed.

### Serum sCD25 levels and clinical extent of LCH disease

**Figure 1:**
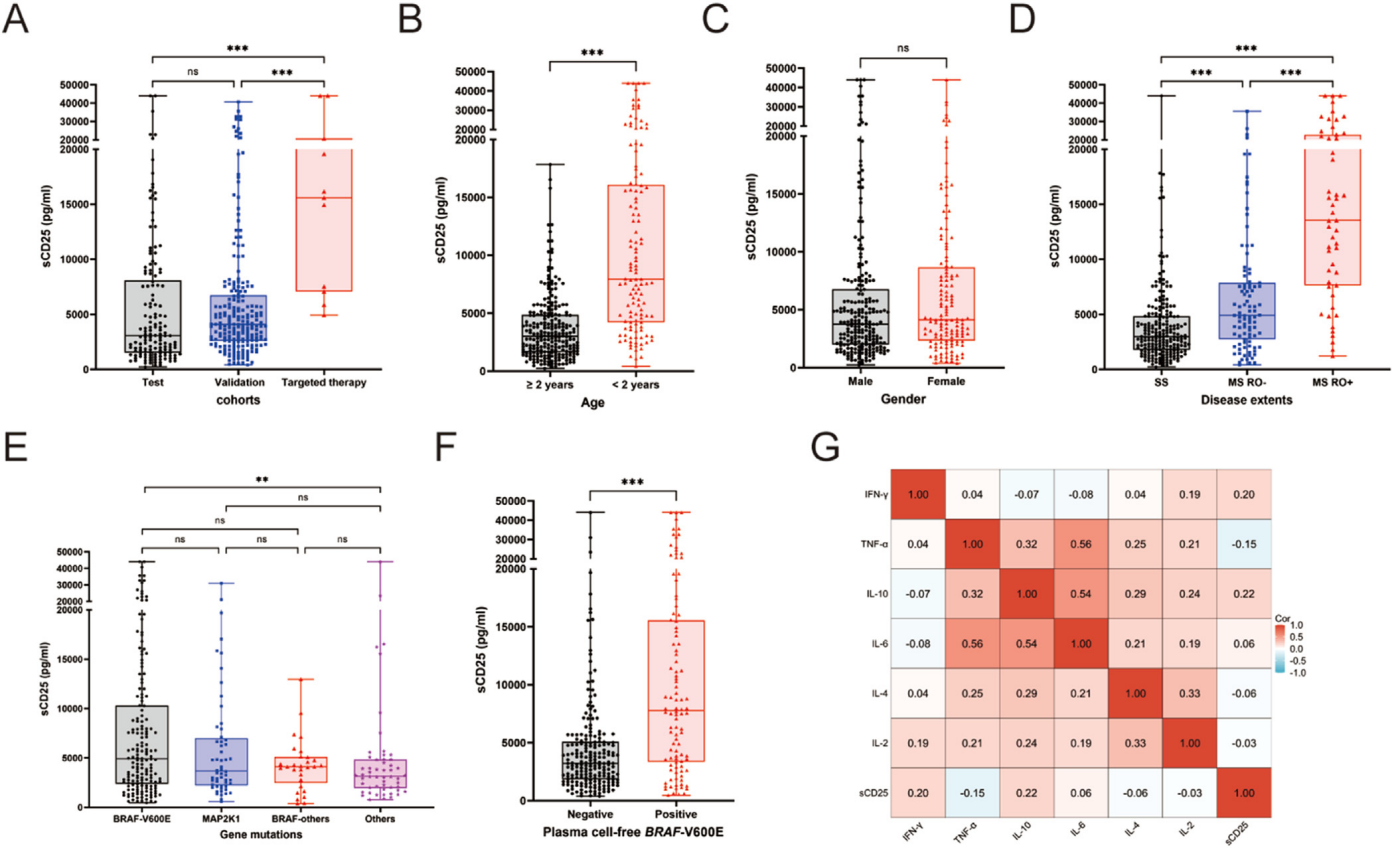
Comparison of serum levels of sCD25 at diagnosis in pediatric Langerhans cell histiocytosis according to clinical-biological characteristics. (A) Patient cohorts. (B) Age. (C) Gender. (D) Disease extents. (E) Gene mutations in tissue lesions. (F) cell-free BRAF-V600E mutations in plasma. (G) Correlation between sCD25 with other cytokines.

The overall median serum level of sCD25 was 3908 pg/ml (ranging from 231 to 44,000 pg/ml). Levels of sCD25 did not differ significantly between the test and validation cohorts, with medians were 3067 and 4097 pg/ml, respectively (*P* = 0.058). sCD25 levels were higher in the dabrafenib group than in either the test or validation cohorts (median: 15,586 pg/ml for the dabrafenib group; both *p* < 0.001; Figure 1A). Patients younger than age two years had higher levels of sCD25 than those older than two years (Figure 1B). There was a significant difference in sCD25 levels across the three disease extents categories, with the highest in MS RO^+^ and the lowest in SS LCH (medians: 13,530 pg/ml for MS RO^+^, 4912 pg/ml for MS, 2979 pg/ml for SS, *p* < 0.001; Figure 1C). Elevated sCD25 levels were associated with the involvements of risk organs, skin, lung, lymph nodes, or pituitary (all *p* < 0.05; Supplementary Figure S2). Intriguingly, sCD25 levels were significantly higher in patients without bone involvement than in those with bone involvement (*p* = 0.0019). Notably, MS RO^+^ patients with macrophage activation syndrome-Hemophagocytic lymphohistiocytosis (MAS-HLH) had higher sCD25 than RO^+^ patients without this syndrome (*p* < 0.001). sCD25 levels did not differ significantly among patients carrying *BRAF*-V600E, *MAP2K1* or *BRAF*-other mutations (Figure 1E). Patients with positive cf*BRAF*-V600E mutations had significantly higher sCD25 levels (*p* < 0.001, Figure 1F).

Furthermore, We found there was only a mild correlation between serum levels of sCD25 and several other cytokines. sCD25 positively correlated with IL-10 (Spearman's *ρ* = 0.22, *p* < 0.001) and IFN-γ (*ρ* = 0.20, *p* < 0.001), but negatively related to TNF-α (*ρ* = −0.15, *p* = 0.003). No correlation was observed between sCD25 levels and IL-6, IL-4, or IL-2 (all *p* values < 0.05; Figure 1G).

### Prognostic significance of sCD25 levels at diagnosis

**Figure 2:**
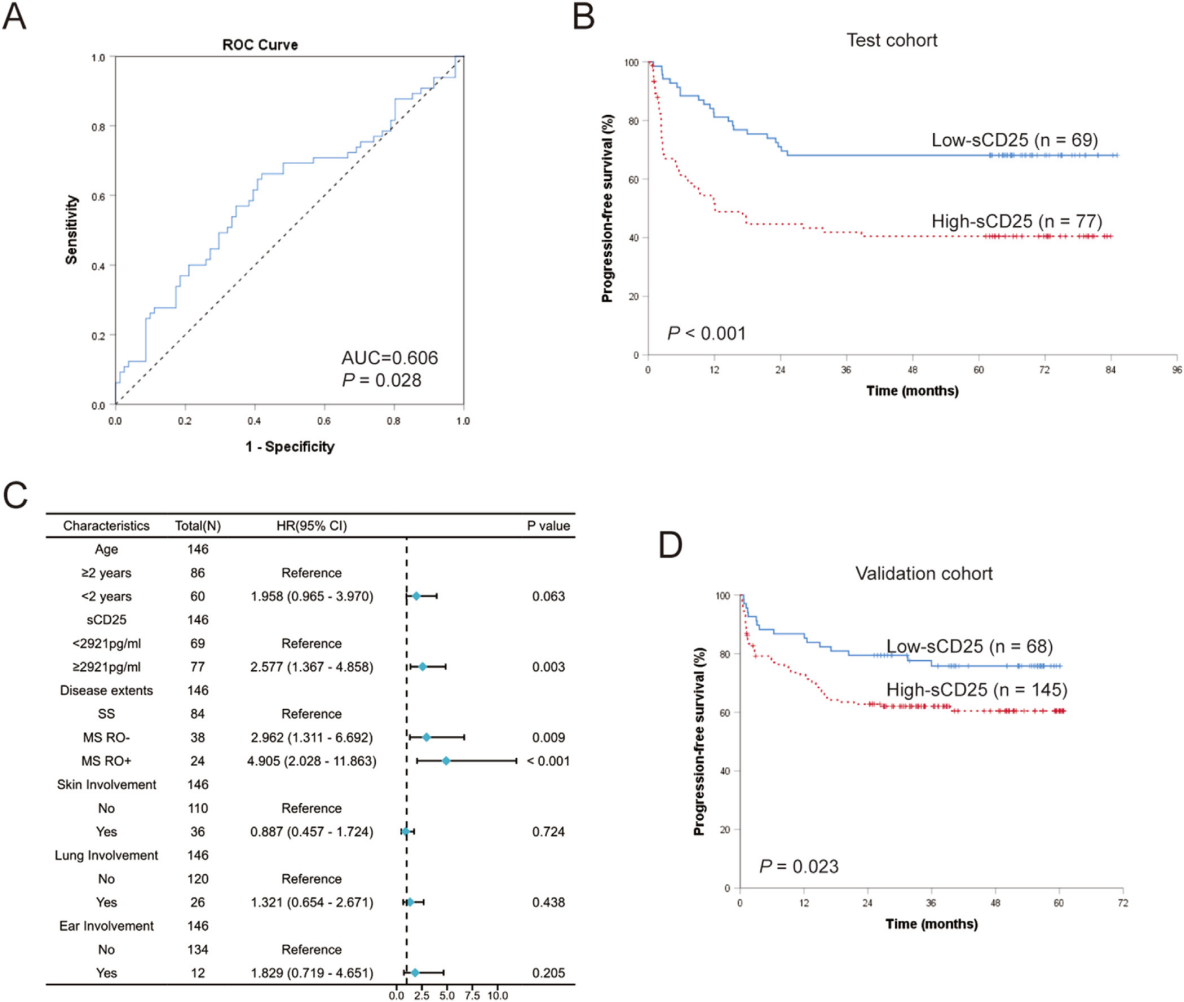
Prognostic significance of serum sCD25 levels at diagnosis in pediatric Langerhans cell histiocytosis (LCH). (A) ROC curve of sCD25 levels for progression and relapse. (B) Comparison of progression-free survival (PFS) for patients in high-sCD25 (≥ 2921 pg/ml) and low-sCD25 group (< 2921 pg/ml) in test cohort. (C) Forest map of multivariate analysis of prognostic risk factors in test cohort. (D) Kaplan–Meier survival curve of patients with high-sCD25 and low-sCD25 in the validation cohort.

To assess the predictive abilities of sCD25 levels for LCH prognosis, we performed ROC curve analysis on 146 patients treated with the first-line treatment from the test cohort. The results revealed that sCD25 could efficiently predict LCH progression and relapse after standard first-line treatment, with an area under the curve (AUC) 0.606 (95 % CI: 0.512–0.701, *p* = 0.028; Figure 2A). An optimal cutoff value for sCD25 was determined to be 2921 pg/ml, with a sensitivity, specificity, PPV, and NPV of 66.2 %, 58.0 %, 55.8 %, and 68.1 %, respectively. According to the optimal cutoff sCD25 value, the authors divided the test cohort into the high-sCD25 group (≥ 2921 pg/ml; *n* = 77) and a low-sCD25 group (< 2921 pg/ml; *n* = 69). Patients in the high-sCD25 group had significantly worse PFS than those in the low-sCD25 group (5-year PFS were 40.4 % ± 5.8 % and 68.1 % ± 5.6 %, respectively, *p* < 0.001; Figure 2B).

We then used univariate analysis to study the effects of baseline characteristics on the PFS of LCH (Supplementary Table S2). The HR for high levels of sCD25 in the test cohort was 2.594 (*p* < 0.001). We also revealed prognostic indicators significantly associated with clinical-biological features, including patients diagnosed before the age of 2 years (HR = 1.839, *p* = 0.014), multisystem disease (HR = 2.930, *p* < 0.001), RO involved (HR = 4.653, *p* < 0.001), the involvements of skin (HR = 2.209, *p* = 0.003), lung (HR = 2.314, *p* = 0.004) and ear (HR = 2.577, *p* = 0.013). In patients assessable for *BRAF* status*,* the presence of *BRAF*-V600E in tissue lesion or plasma was associated with a worse prognosis and increased risk of recurrence/progression; the HRs were 1.973 (*p* = 0.029) and 3.137 (*p* < 0.001), respectively.

Clinical characteristics associated with an increased prognostic risk in univariate analysis (*p* < 0.05) and sCD25 levels grouping were subjected to multivariate Cox regression analysis to identify independent prognostic factors. *BRAF*-V600E mutation status was not included in the multivariate analysis due to missing data for about one-third of the patients. According to the multivariate analysis, multisystem disease, RO involvements, and high levels of sCD25 were confirmed as independent prognostic factors for PFS in LCH patients (Figure 2C). Patients with high sCD25 levels had an increased risk of progression or relapse compared to those with low sCD25 levels (HR = 2.577, *p* = 0.003). RO involvement was found to be the strongest independent poor prognostic factor (HR = 4.905, *p* < 0.001).

Furthermore, we validated the prognosis impact of sCD25 levels in the independent validation cohort. When the 213 treated LCH patients in the validation cohort were assigned into the two subgroups based on the cut-off level of sCD25 (2921 pg/ml), analysis of survivals also showed poorer PFS in the high-sCD25 group compared to the low-sCD25 group (3-year PFS was 60.4 % ± 4.3 % and 75.8 % ± 5.3 %, respectively; *p* = 0.023; Figure 2D). The HR for high-sCD25 patients in the validation cohort was 1.891 (*p* = 0.025).

### Prevalence of sCD25 grouping among patients according to clinical features

Grouping of CD25 levels according to the cut-off values was related to patient clinical and biological characteristics (Supplementary Figure S3). High sCD25 level was more prevalent in patients younger than two years of age or with RO involvement. Patients in the high-sCD25 group had more multisystem involvements and were more likely to have involvement of skin, lung, or lymph nodes. The frequency of sCD25 subgroups did not significantly differ among *BRAF* or *MAP2K1* gene subtypes, but high sCD25 level was closely associated with the positivity of cf*BRAF*-V600E in plasma.

### Significance of sCD25 in subgroups

We analyzed the prognostic significance of sCD25 levels in the three disease extent category subgroups. However, the results of ROC analysis were not statistically significant in each subgroup of both cohorts (Supplementary Table S3), which may be due to the relatively small number of cases in each subgroup and the uneven distribution of events. We attempted to set specific sCD25 thresholds for each patient subgroup in the test cohort using maximum Youden's index. The cut-off value was higher for MS RO^+^ LCH compared to MS RO^−^or SS LCH. The univariate analysis showed that MS RO^+^ patients with high sCD25 levels (≥ 4874pg/ml) had a significantly increased risk of recurrence/progression (HR= 8.731, *p* = 0.039). However, these differences were insignificant in the validation cohort. A larger sample of cases is needed to draw a definitive conclusion.

### Risk scoring model

**Figure 3:**
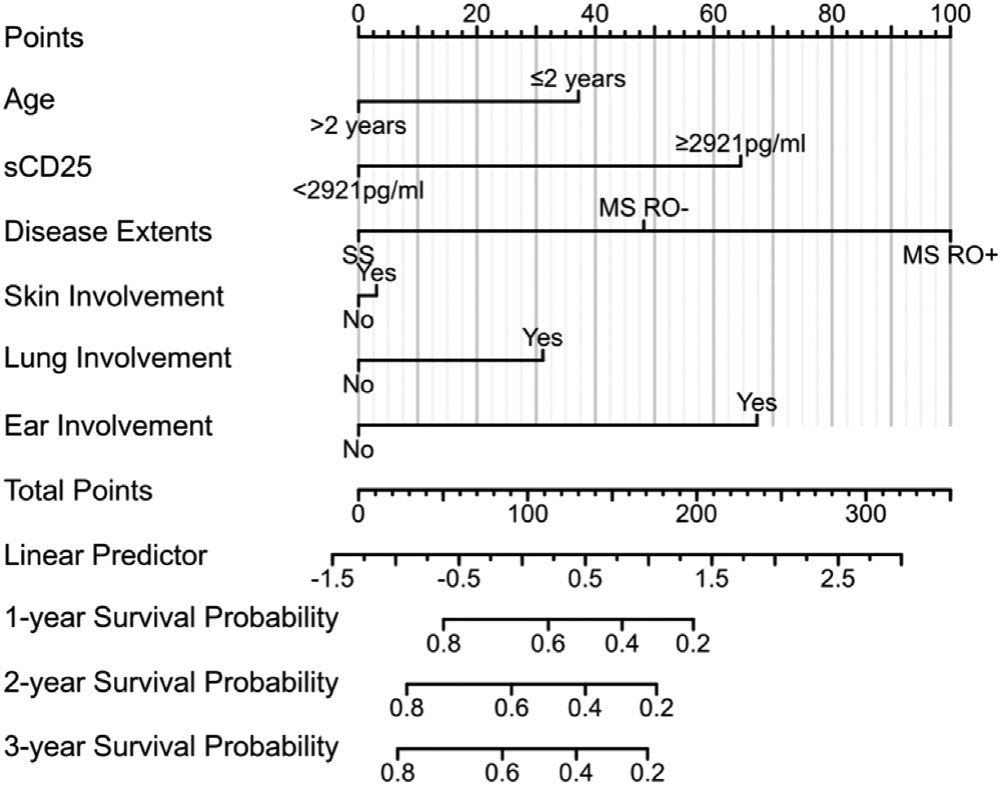
A nomogram from the risk scoring model that integrates sCD25 levels and other clinical risk factors.

We developed a risk-scoring model that integrates sCD25 levels and other clinical risk factors from the Cox regression analysis of the test cohort. A nomogram was constructed based on the model (Figure 3), with a C-index of 0.708 (95 % CI:0.673–0.742), confirming its prognostic predictive power. We validated the nomogram in the validation cohort, where the C-index was 0.764 (95 % CI: 0.734–0.795).

## Discussion

This study investigated the association between sCD25 levels and the clinical characteristics and prognosis of pediatric LCH, demonstrating that the higher levels of sCD25 were associated with the high-risk features and inferior outcomes of LCH.

LCH exhibits characteristics of both neoplasia and immune activation, with inflammation playing a vital role in its pathophysiology.^24^ LCH lesions are accompanied by a diverse inflammatory infiltrate, enriching dysfunctional T cells.^25^ The IL-2 pathway plays a crucial role in regulating the immune response, and sCD25, a component of the IL-2 receptor, is shed during immune activation that serves as a marker of T cell activation.^26^^,^^27^ Elevated sCD25 was previously reported in LCH patients before treatment and correlated with disease extent.^28^ The present finding showed that sCD25 levels were significantly higher in patients with multisystem disease and RO involvements, in agreement with the above reports. Moreover, we demonstrated that the higher sCD25 levels at baseline were closely associated with other high-risk features, including younger age and involvements of skin, lung, or lymph nodes. Increased sCD25 also correlated with the positivity of cf*BRAF*-V600E in plasma, which has been linked to inferior prognosis. Notably, the data showed remarkably elevated sCD25 levels in LCH patients with MAS-HLH, a life-threatening condition characterized by the over-activation of T cells and macrophages, leading to excessive production of inflammatory cytokines, cytopenias, hepatosplenomegaly, and many other manifestations.^29^ sCD25 is mainly produced by activated T cells, indicating up-regulated levels of sCD25 in LCH patients who had activated T cell proliferation.

Advances in risk-stratified treatment and the application of targeted therapies have significantly improved the overall survival of LCH patients. However, refractory or recurrent diseases remains a major challenge in further improving prognosis, with approximately one-third of patients relapsing after discontinuation of therapy.^19^^,^^30^ The present study demonstrates that the high sCD25 levels at diagnosis independently predict inferior PFS in patients receiving first-line chemotherapy, presenting cut-off values, sensitivity, specificity, and accuracy. sCD25 levels ≥ 2921 pg/ml were found to have an independent predictive impact (hazard ratio: 2.577) in the test cohort, which was confirmed in the independent validation cohort. Due to inter-laboratory deviations, the optimal cut-off for sCD25 varied among different laboratories based on their specific reference values. In particular, measurements should be standardized to minimize inter-laboratory variability.

## Conclusion

The present results demonstrated that elevated serum sCD25 at diagnosis in pediatric LCH patients was associated with high-risk clinical features and worse prognosis. The data revealed that baseline sCD25 levels had predictive value for progression/recurrence of LCH following treatment with first-line chemotherapy. These findings highlight the need for prospective, independent validation in larger patient cohorts to confirm the predictive utility and better define the potential clinical utility. Exploration of such inflammatory biomarkers is valuable in elucidating the role of inflammation in LCH disease pathogenesis and progression, further refining clinical stratification, and developing new therapeutic targets.

## Funding

This work was supported by the 10.13039/501100004826Beijing Natural Science Foundation [No. 7242053 and 7232058], Capital's Funds for Health Improvement and Research [No.2022-2-1141], National Natural Science Foundation of China [No.82141119 and No.82070202], and Funding for Reform and Development of Beijing Municipal Health Commission.

## Conflicts of interest

The authors declare no conflicts of interest.

## Appendix Supplementary materials

Image, application 1

## Footnotes

Supplementary material associated with this article can be found in the online version at doi:10.1016/j.jped.2024.08.005.

## References

[1] Rodriguez-Galindo C., Allen C.E. (2020). Langerhans cell histiocytosis. Blood.

[2] Gulati N., Allen C.E. (2021). Langerhans cell histiocytosis: version 2021. Hematol Oncol.

[3] Durham B.H., Lopez Rodrigo E., Picarsic J., Abramson D., Rotemberg V., de Munck S. (2019). Activating mutations in CSF1R and additional receptor tyrosine kinases in histiocytic neoplasms. Nat Med.

[4] Badalian-Very G., Vergilio J.A., Degar B.A., MacConaill L.E., Brandner B., Calicchio M.L. (2010). Recurrent BRAF mutations in Langerhans cell histiocytosis. Blood.

[5] Allen C.E., Longo D.L., Merad M., McClain K.L. (2018). Langerhans-cell histiocytosis. N Engl J Med.

[6] Haroche J., Cohen-Aubart F., Rollins B.J., Donadieu J., Charlotte F., Idbaih A. (2017). Histiocytoses: emerging neoplasia behind inflammation. Lancet Oncol.

[7] Allen C.E., Ladisch S., McClain K.L. (2015). How I treat Langerhans cell histiocytosis. Blood.

[8] Tazi A., Moreau J., Bergeron A., Dominique S., Hance A.J., Soler P. (1999). Evidence that Langerhans cells in adult pulmonary Langerhans cell histiocytosis are mature dendritic cells: importance of the cytokine microenvironment. J Immunol.

[9] Cai F., Peng Z., Xu H., Gao H., Liao C., Xu X. (2023). Immune microenvironment associated with the severity of Langerhans cell histiocytosis in children. Cytokine.

[10] Morimoto A., Oh Y., Nakamura S., Shioda Y., Hayase T., Imamura T. (2017). Inflammatory serum cytokines and chemokines increase associated with the disease extent in pediatric Langerhans cell histiocytosis. Cytokine.

[11] Mitchell J.M., Berzins S.P., Kannourakis G. (2018). A potentially important role for T cells and regulatory T cells in Langerhans cell histiocytosis. Clin Immunol.

[12] Liao W., Lin J.-X., Leonard W.J. (2011). IL-2 family cytokines: new insights into the complex roles of IL-2 as a broad regulator of T helper cell differentiation. Curr Opin Immunol.

[13] Damoiseaux J. (2020). The IL-2 - IL-2 receptor pathway in health and disease: the role of the soluble IL-2 receptor. Clin Immunol.

[14] Cao S., Liu X., Li Y., Yang Y., Cai X., Cong S. (2024). Serum sCD25 is an indicator for rheumatoid arthritis-associated interstitial lung disease. Clin Exp Rheumatol.

[15] Siemiatkowska A., Bryl M., Kosicka-Noworzyn K., Tvrdon J., Golda-Gocka I., Barinow-Wojewodzki A. (2021). Serum sCD25 protein as a predictor of lack of long-term benefits from immunotherapy in non-small cell lung cancer: a pilot study. Cancers (Basel).

[16] Mostafa G.A., Ibrahim H.M., Al Sayed S.A., Gendy Y.G., Aly D.M., Shousha G.A (2022). Up-regulated serum levels of soluble CD25 and soluble CD163 in pediatric patients with SARS-CoV-2. Eur J Pediatr.

[17] Rosso D.A., Roy A., Zelazko M., Braier J.L. (2002). Prognostic value of soluble interleukin 2 receptor levels in Langerhans cell histiocytosis. Br J Haematol.

[18] Haupt R., Minkov M., Astigarraga I., Schäfer E., Nanduri V., Jubran R. (2013). Langerhans cell histiocytosis (LCH): guidelines for diagnosis, clinical work-up, and treatment for patients till the age of 18 years. Pediatr Blood Cancer.

[19] Cui L., Wang C.J., Lian H.Y., Zhang L., Ma H.H., Wang D. (2023). Clinical outcomes and prognostic risk factors of Langerhans cell histiocytosis in children: results from the BCH-LCH 2014 protocol study. Am J Hematol.

[20] Yang Y., Wang D., Cui L., Ma H.-H., Zhang L., Lian H.-Y. (2021). Effectiveness and safety of Dabrafenib in the treatment of 20 Chinese children with BRAFV600E-Mutated Langerhans cell histiocytosis. Cancer Res Treat.

[21] Zhao X.X., Lian H.Y., Zhang L., Ma H.H., Wang D., Zhao Y.Z. (2022). Significance of serum Th1/Th2 cytokine levels in underlying disease classification of childhood HLH. Cytokine.

[22] Wang C.J., Cui L., Li S.S., Ma H.H., Wang D., Lian H.Y. (2024). Genetic landscape and its prognostic impact in children with Langerhans cell histiocytosis. Arch Pathol Lab Med.

[23] Cui L., Zhang L., Ma H.H., Wang C.J., Wang D., Lian H.Y. (2020). Circulating cell-free BRAF V600E during chemotherapy is associated with prognosis of children with Langerhans cell histiocytosis. Haematologica.

[24] Lee J.W., Shin H.Y., Kang H.J., Kim H., Park J.D., Park K.D. (2014). Clinical characteristics and treatment outcome of Langerhans cell histiocytosis: 22 years’ experience of 154 patients at a single center. Pediatr Hematol Oncol.

[25] Sengal A., Velazquez J., Hahne M., Burke T.M., Abhyankar H., Reyes R. (2021). Overcoming T-cell exhaustion in LCH: PD-1 blockade and targeted MAPK inhibition are synergistic in a mouse model of LCH. Blood.

[26] Spolski R., Li P., Leonard W.J. (2018). Biology and regulation of IL-2: from molecular mechanisms to human therapy. Nat Rev Immunol.

[27] Zhou P. (2022). Emerging mechanisms and applications of low-dose IL-2 therapy in autoimmunity. Cytokine Growth Factor Rev.

[28] Wang W., Liu X., Yang S., Peng X., Ma Y., Xiong X. (2023). Serum levels of soluble interleukin 2 receptor (sIL-2R), tumor necrosis factor-alpha (TNF-alpha), and immunoglobulin M are correlated with the disease extent in childhood Langerhans cell histiocytosis. J Cancer Res Clin Oncol.

[29] Wang D., Chen X.H., Wei A., Zhou C.J., Zhang X., Ma H.H. (2022). Clinical features and treatment outcomes of pediatric Langerhans cell histiocytosis with macrophage activation syndrome-hemophagocytic lymphohistiocytosis. Orphanet J Rare Dis.

[30] Rigaud C., Barkaoui M.A., Thomas C., Bertrand Y., Lambilliotte A., Miron J. (2016). Langerhans cell histiocytosis: therapeutic strategy and outcome in a 30-year nationwide cohort of 1478 patients under 18 years of age. Br J Haematol.

