# Supplementary material for: The clinical impact of serum soluble CD25 levels in children with Langerhans cell histiocytosis
Source: J Pediatr (Rio J). 2024 Sep 10;101(2):194–201. doi: 10.1016/j.jped.2024.08.005 (PMC11889682; doi:10.1016/j.jped.2024.08.005)
Supplement: Supplementary file 1 [file mmc1.docx]

**JPED-D-24-00244 – Supplementary Material**

**Supplementary Table S1** Genes and fusion genes in panel used to targeted sequencing.

| AKT1 | CDKN2B | FGFR1 | KIF5B | MAP4K4 | NR4A2 | RHOA | TET2 |
| --- | --- | --- | --- | --- | --- | --- | --- |
| ALK | CHEK1 | FGFR2 | KIT | MAPK1 | NRAS | RIPK1 | TGFB1 |
| ANK3 | CHEK2 | FIP1L1 | KLLN | MAPK10 | NTRK1 | RNF11 | TGFBR1 |
| APC | CLIP2 | FLT3 | KMT2D | MAPK14 | PACSIN2 | ROCK1 | TNF |
| ARAF | CREBBP | FOXO1 | KRAS | MAPK3 | PAK1 | RORC | TNFAIP3 |
| ARHGAP1 | CRK | FOXO3 | LMNA | MAPK7 | PAK2 | ROS1 | TP53 |
| ARID1A | CSF1R | GAB1 | MAP2K1 | MAPK8 | PDGFRA | SETD2 | TRIM28 |
| ARID1B | CSF3R | GATA3 | MAP2K2 | MAPK9 | PDK1 | SF3B1 | TSC1 |
| ARID2 | CSHL1 | GATA4 | MAP2K3 | MAPKAPK2 | PICK1 | SH2D2A | TSC2 |
| ATM | CTBP1 | GCK | MAP2K4 | MDM2 | PIK3CA | SIRT1 | TWIST1 |
| ATR | CTBP2 | GNA11 | MAP2K5 | MET | PIK3CB | SMAD2 | ULK1 |
| B2M | CTNNA1 | GNAQ | MAP2K6 | MIGA1 | PIK3CD | SMAD3 | USP8 |
| B9D2 | CXCL8 | GRB2 | MAP2K7 | MKNK1 | PIK3CG | SMAD4 | VCAN |
| BAG3 | CYFIP2 | GSK3A | MAP3K1 | MST1 | PIK3R1 | SMO | VCL |
| BCAR1 | DAXX | GSK3B | MAP3K10 | MTAP | PIK3R2 | SNAI1 | VCP |
| BRAF | DDR2 | HRAS | MAP3K11 | MTOR | PRKCE | SNAI2 | WNT1 |
| BTK | DNMT3A | HSPB8 | MAP3K12 | MUC4 | PTEN | SOS1 | ZBTB42 |
| CASP8AP2 | DOCK1 | HSPD1 | MAP3K13 | MYC | PTK2 | SOS2 | ZEB1 |
| CBL | EDNRA | IKBKB | MAP3K14 | MYD88 | PTPN11 | SPRED1 | ZEB2 |
| CCND1 | EGFR | IKZF1 | MAP3K2 | MYH9 | PTPN3 | SQSTM1 | CLIP2-BRAF |
| CCND2 | EGR1 | IL17A | MAP3K20 | NF1 | RAC1 | SRC | FIP1L1-PDGFRA |
| CCND3 | EGR2 | IL1A | MAP3K21 | NF2 | RAF1 | STAT1 | KIF5B-ALK |
| CCR6 | EIF4EBP1 | IL1RN | MAP3K3 | NFE2L2 | RALA | STAT3 | LMNA-NTRK1 |
| CD79B | EP300 | IL6 | MAP3K4 | NFKB1 | RALGDS | STAT5B | MIGA1-BRAF |
| CDC25A | EPHA6 | IRF2BP2 | MAP3K5 | NFKB2 | RASA1 | TAB1 | PACSIN2-BRAF |
| CDC42 | ERBB2 | IRF8 | MAP3K6 | NFKBIA | RB1 | TAB2 | RNF11-BRAF |
| CDKN1A | ERBB3 | JAK1 | MAP3K7 | NLK | RBBP6 | TAF1 | IRF2BP2-NTRK1 |
| CDKN1B | EXTL3 | JAK2 | MAP3K8 | NOTCH1 | RELA | TAOK1 | EPHA6-RET |
| CDKN1C | EZH2 | JAK3 | MAP3K9 | NOTCH2 | RET | TAOK2 | FAM73A-BRAF |
| CDKN2A | FBXW7 | KIF14 | MAP4K1 | NR2C2 | RHEB | TERT |  |

**Supplementary Table S2** Univariate analysis of prognostic factors for progression-free survival in children with Langerhans cell histiocytosis in the test cohort

| Characteristics | Total  n (%) | Univariate analysis  Hazard ratios (95% CI) | *P-* value |
| --- | --- | --- | --- |
| Total | 146 | – | – |
| sCD25 levels |  |  |  |
| Low (< 2921 pg/ml) | 69 (47.3) | 1 | < 0.001 |
| High (≥ 2921 pg/ml) | 77 (52.7) | 2.594 (1.550 – 4.343) |  |
| Gender |  |  |  |
| Male | 88 (60.3) | 1 | 0.591 |
| Female | 58 (39.7) | 1.145 (0.699 – 1.875) |  |
| Age at diagnosis |  |  |  |
| ≥ 2 years | 86 (58.9) | 1 | 0.014 |
| < 2 years | 60 (41.1) | 1.839 (1.130 – 2.994) |  |
| Disease extents |  |  |  |
| SS | 84 (57.5) | 1 |  |
| MS RO^-^ | 38 (26) | 2.930 (1.678 – 5.116) | < 0.001 |
| MS RO^+^ | 24 (16.4) | 4.653 (2.452 – 8.828) | < 0.001 |
| Involvements |  |  |  |
| Bone |  |  |  |
| No | 18 (12.3) | 1 | 0.965 |
| Yes | 128 (87.7) | 0.983 (0.469 – 2.062) |  |
| Skin |  |  |  |
| No | 110 (75.3) | 1 | 0.003 |
| Yes | 36 (24.7) | 2.209 (1.311 – 3.723) |  |
| Liver |  |  |  |
| No | 125 (85.6) | 1 | 0.001 |
| Yes | 21 (14.4) | 2.835 (1.540 – 5.219) |  |
| Spleen |  |  |  |
| No | 133 (91.1) | 1 | < 0.001 |
| Yes | 13 (8.9) | 3.917 (1.917 – 8.003) |  |
| Hematologic |  |  |  |
| No | 135 (92.5) | 1 | < 0.001 |
| Yes | 11 (7.5) | 4.782 (2.154 – 10.618) |  |
| Lung |  |  |  |
| No | 120 (82.2) | 1 | 0.004 |
| Yes | 26 (17.8) | 2.314 (1.298 – 4.125) |  |
| Lymph nodes |  |  |  |
| No | 131 (89.7) | 1 | 0.096 |
| Yes | 15 (10.3) | 1.878 (0.894 – 3.942) |  |
| Pituitary |  |  |  |
| No | 138 (94.5) | 1 | 0.094 |
| Yes | 8 (5.5) | 2.186 (0.874 – 5.463) |  |
| Eye |  |  |  |
| No | 134 (91.8) | 1 | 0.361 |
| Yes | 12 (8.2) | 0.048 (0 – 32.755) |  |
| Ear |  |  |  |
| No | 142 (97.3) | 1 | 0.013 |
| Yes | 4 (2.7) | 2.577 (1.224 – 5.424) |  |
| *BRAF*-V600E mutations in Tissue lesions | |  |  |
| Evaluable patients | 105 |  |  |
| Negative | 43 (41.0) | 1 | 0.029 |
| Positive | 62 (59.0) | 1.973 (1.071 – 3.634 ) |  |
| *BRAF*-V600E in plasma cell-free DNA | |  |  |
| Evaluable patients | 119 |  |  |
| Negative | 72 (60.5) | 1 | < 0.001 |
| Positive | 47 (39.5) | 3.137 (1.832 – 5.374) |  |

**Supplementary Table S3** Prognostic significance of sCD25 levels in three disease extents category subgroups.

| Groups | n | | Median  (Interquartile range) | ROC analysis | | | Univariate analysis | | |
| --- | --- | --- | --- | --- | --- | --- | --- | --- | --- |
|  | Total | Progression/  Relapse |  | AUC | *P*-value | Cut-off | HR | 95% CI | *P*-value |
| Test cohort |  |  |  |  |  |  |  |  |  |
| SS LCH | 84 | 26 | 2088  (1274-3738) | 0.506 | 0.931 | 2921 | 1.499 | 0.688-3.265 | 0.308 |
| MS RO- LCH | 38 | 24 | 4072  (1878-4072) | 0.542 | 0.672 | 3134 | 2.341 | 0.966-5.675 | 0.060 |
| MS RO+ LCH | 24 | 15 | 10165  (4935-15182) | 0.607 | 0.387 | 4874 | 8.731 | 1.114-68.422 | 0.039 |
| Validation cohort |  |  |  |  |  |  |  |  |  |
| SS LCH | 140 | 27 | 3548  (2249-5077) | 0.506 | 0.928 | 2921 | 1.493 | 0.653-3.414 | 0.342 |
| MS RO- LCH | 53 | 25 | 4912  (3163-7470) | 0.624 | 0.121 | 3134 | 2.513 | 1.080-5.844 | 0.032 |
| MS RO+ LCH | 20 | 19 | 20833  (11575-31220) | 0.211 | 0.340 | 4874 | 1.968 | 0.623-6.219 | 0.249 |


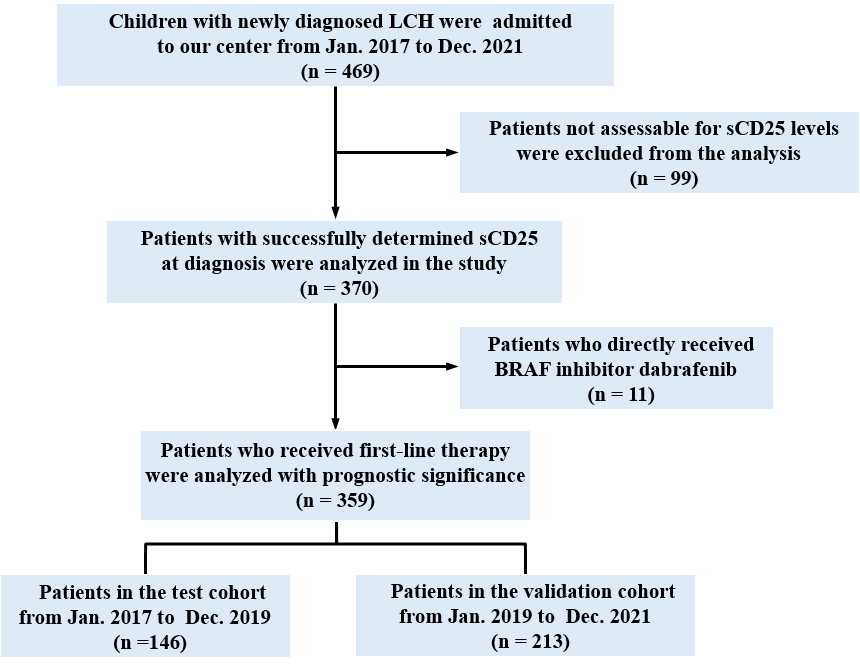


**Supplementary Figure S1** Flow diagram for the study patients.


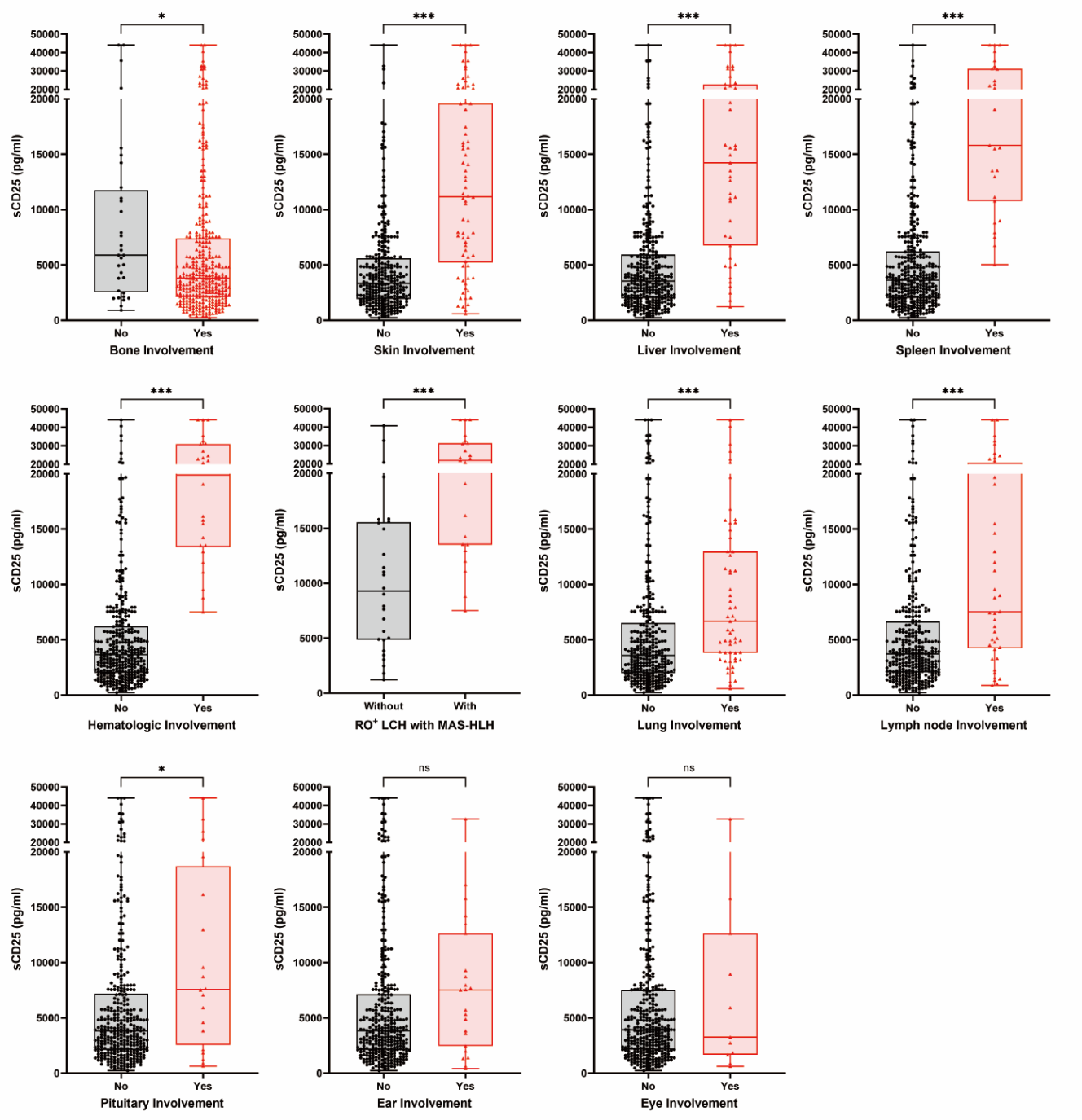


**Supplementary Figure S2** Correlation between CD25 levels and organs involvements.


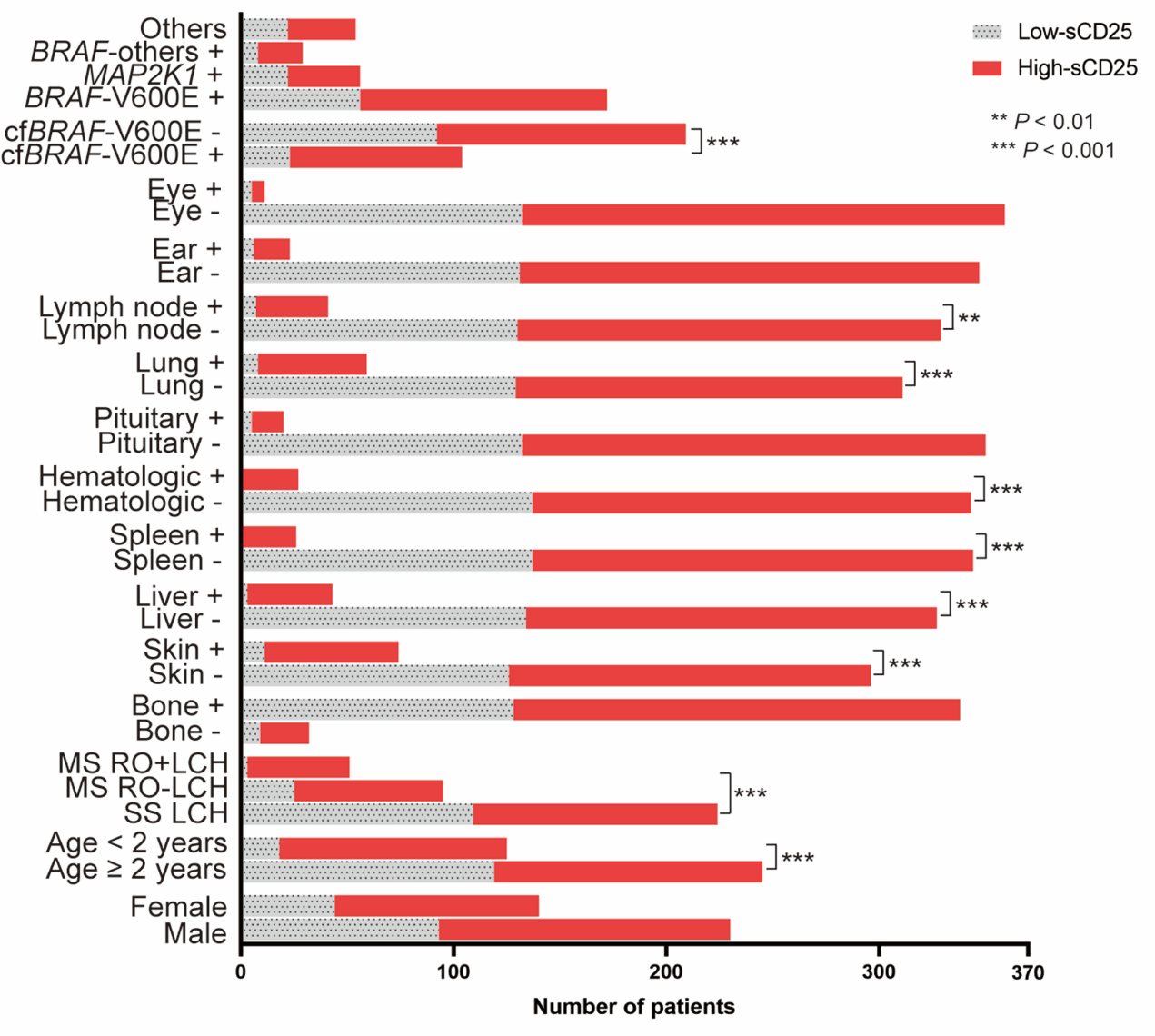


**Supplementary Figure S3** Prevalence of sCD25 grouping among patients according to the clinical-biological features.
